# The sphenopalatine artery: a surgical challenge in epistaxis

**DOI:** 10.1590/S1808-86942012000400009

**Published:** 2015-10-20

**Authors:** Gustavo Lara Rezende, Vitor Yamashiro Rocha Soares, Waldete Cabral Moraes, Carlos Augusto Costa Pires de Oliveira, Márcio Nakanishi

**Affiliations:** MSC at the University of Brasilia (ENT at the Hospital de Base do Distrito Federal); MD, ENT at the Brasilia University Hospital (Fellow on ENT at the Brasilia University Hospital); MD, Pathologist (Head of the Pathology Department of the Hospital de Base do Distrito Federal); Post-Doctoral degree at the Harvard Medical School (Adjunct Professor and Head of the Otorhinolaryngology and Head and Neck Surgery Department of the University of Brasília); PhD in Otorhinolaryngology at the Medical School of the University of São Paulo (Adjunct Professor at the Otorhinolaryngology and Head and Neck Surgery Department of the University of Brasília; ENT at the Distirto Federal Secretary of Health). Universidade de Brasília

**Keywords:** epistaxis, maxillary artery, nose

## Abstract

Knowledge on the anatomy of the sphenopalatine artery (SPA) and its branches is fundamental for the success of the endoscopic treatment of posterior epistaxis. However, the complex anatomical variations seen in the irrigation of the nasal cavity poses a significant surgical challenge.

**Objective**: This paper aims to describe the endoscopic anatomy of the SPA in human cadavers.

**Materials and Methods**: This is a contemporary cross-sectional cohort study carried out between April 2010 and August 2011. The presence of the ethmoidal crest on the lamina perpendicular to the palatine bone and the location of the principal sphenopalatine foramen (PSF) and the accessory sphenopalatine foramen (ASF) were analyzed in 28 cadavers, and the branches emerging from the foramens were counted.

**Results**: Fifty-six nasal fossae were analyzed. The ethmoidal crest was present in 96% of the cases and was located anteriorly to the PSF in most cases. The PSF was located in the transition area between the middle and the superior meatus in all cases. The ASF was seen in 12 cases. Most nasal fossae (n = 12) presented a single bilateral arterial trunk emerging from the PSF. In other cases, three (n = 8) or two (n = 5) arterial trunks emerged bilaterally from the PSF. In most cases, the SPA emerged as a single trunk from the ASP.

**Conclusions**: The anatomy of the SPA is highly variable. The success of the treatment for severe epistaxis relies heavily on adequate knowledge of the possible anatomical variations of the sphenopalatine artery.

## INTRODUCTION

The main source of blood in the nasal cavity is the sphenopalatine artery (SPA), a branch of the external carotid system. The SPA is situated in the posterior region of the nasal cavity, and is involved in most severe epistaxis episodes^1^, ^2^. Failure rates in cauterizing or ligating the SPA in severe nosebleeds range between 2% to 10%^3^. Even in experienced hands, the complex anatomical variations of nasal cavity vessels pose a significant challenge.

The sphenopalatine foramen is a notch on the upper margin of the palatine bone between the orbit and the sphenopalatine process. It turns into a foramen as the palatine bone joins the sphenoid bone on the lateral nasal wall. Variations in size, shape, location, and number of branches emerging from its orifice have been described scarcely in the literature^4^, ^5^. Studies are required to better define the anatomical endoscopic landmarks and increase the effectiveness of epistaxis surgical treatments.

Current trends dictate that posterior packing should be replaced by the endoscopic ligation of the sphenopalatine artery in cases of posterior bleeding to reduce morbidity and patient discomfort levels^6^. To that end, mastery of the endoscopic surgical technique and profound knowledge of the nose's vascularization are required for the success of the treatment. However, endoscopic visualization changes the way in which these structures are identified. This is not to say that the anatomy is changed, but only how this anatomy is seen from the endoscope.

This study aims to describe the endoscopic anatomy of the sphenopalatine artery in cadavers and the possible anatomic variations, and assess the bone landmarks used to identify the sphenopalatine foramen.

## MATERIALS AND METHODS

A descriptive anatomic study on the sphenopalatine artery was performed at the Pathology Service of a tertiary care hospital between April of 2010 and August of 2011. Twenty-eight cadavers were included in the study. Specimen collection took place between three and 12 hours as of the time of death of the subjects. The data collected on the cadavers included race, gender, age, time of death, time of autopsy, and cause of death as per the autopsy protocol of the hospital's pathology service. Cadavers with nose trauma, previous nose surgery, or nasal diseases preventing dissection were excluded.

The analysis of the endoscopic anatomy of the sphenopalatine artery was carried out using a nasal endoscope with a 30-degree rigid scope, 4.0 mm (Karl Storz), fiber optics, and a Komlux light source (250 watts), a Toshiba monitor (model CRT 1030), and a video endoscopy device (model Toshiba IK-CU44A). Images were recorded and stored using a video capturing software (Pinnacle Systems, Inc., Studio Movie Box HD).

All dissections were done bilaterally, in accordance with the following steps of nose endoscopic surgery: (1) The middle concha was shifted medially to expose, with the aid of an endonasal probe, the transition between the posterior medial maxillary wall and the perpendicular portion of the palatine bone; one vertical incision was made on the mucosa of the lateral nasal wall of 1.5 cm from the beginning of the perpendicular portion of the palatine bone to the upper portion of the inferior concha; (2) A mucoperiosteal flap was made along the posterior area of the nose, from the transition between the middle and posterior meatus, until the sphenopalatine foramen and its vessels were identified; (3) Dissection was done until the anterior wall of the sphenoid sinus to identify other possible arterial branches. Samples of one centimeter starting from the point of emergence of the foramen were collected from the best segments bilaterally. These samples were taken to histopathology for testing to validate the arterial origin of the visualized structure.

The anatomic structures were identified and categorized for: (1) Presence of anterior ethmoidal spine on the perpendicular plate of the palatine bone; (2) Location and presence of the sphenopalatine foramen (SPF) and the accessory SPF (ASPF). The SPF was considered as the largest bony orifice on the lateral nasal wall from which the arterial trunks adjacent to the ethmoid crest of the lamina perpendicular to the palatine bone. The ASPF was described as a smaller bony orifice beyond the SPF; (3) Number of arterial branches stemming from the identified foramens; and (4) Prevalence and analysis of symmetry (presence and location of the SPF and the ASPF, and number of arterial branches stemming from the SPF and the ASPF).

The locations of the sphenopalatine foramen and the accessory sphenopalatine foramen were defined in relation to the insertion point of the middle concha: a) on the superior meatus (SM): the opening of the SPF appears above the middle concha insertion point; b) on the transition between the superior and middle meatus (SM/MM): the opening of the SPF occurs under the ethmoidal spine; c) on the middle meatus (MM): the opening of the SPF is below the line of insertion of the middle concha.

This study was approved by the local Ethics Committee and given permit $n^{\underline{o}}$ 36/2010.

## RESULTS

Fifty-six nasal fossae of 28 cadavers were analyzed, 13 of which males and 15 females. Mean age was 58.32 ± 17.17 years, and ages ranged between 11 and 91. Fifteen were Caucasians and 13 were of African descent. (Table 1).

**Table 1:** Sample demographics.

Variable	n	%
Age		
< 50 years	6	21.4
51 to 60 years	11	39.3
> 60 years	11	39.3
Gender		
Female	15	53.6
Male	13	46.4
Race		
Caucasian	15	53.6
Black	13	46.4

The ethmoidal spine was present in 96.4% of the cases (Table 2). Only one of the cadavers did not have an ethmoidal spine. In most of the cases it appeared on both sides and anteriorly to the SPF (n = 24) and only once on both sides and posteriorly to the SPF, bilaterally and superiorly to SPF and unilaterally and to the left of the SPF. In all cases (n = 28) the sphenopalatine foramen was located in the transition between the middle meatus and the superior meatus. The accessory sphenopalatine foramen was present in 12 cases. Locations can be seen on Table 3.

**Table 2:** Location of the ethmoid crest of the perpendicular lamina of the palatine bone in absolute and percent values.

Variable	n	%
Ethmoid crest		
Bilateral Anterior	24	85.7
Bilateral Posterior	1	3.6
Bilateral Superior	1	3.6
Unilateral to the left	1	3.6
Absent	1	3.6

**Table 3:** Relative location of the sphenopalatine (SPF) and accessory foramens (ASPF).

Sphenopalatine foramen (SPF)	n	%
Middle/Superior Meatus	28	100
Middle Meatus	0	-
Superior Meatus	0	-
Accessory sphenopalatine foramen (ASPF) location	N	%
One, to the right of the middle meatus	4	33.3
One, to the left of the middle meatus	4	33.3
One, on the left superior meatus	1	8.3
Bilateral, on the left superior meatus	3	24.9

**Figure 1:**
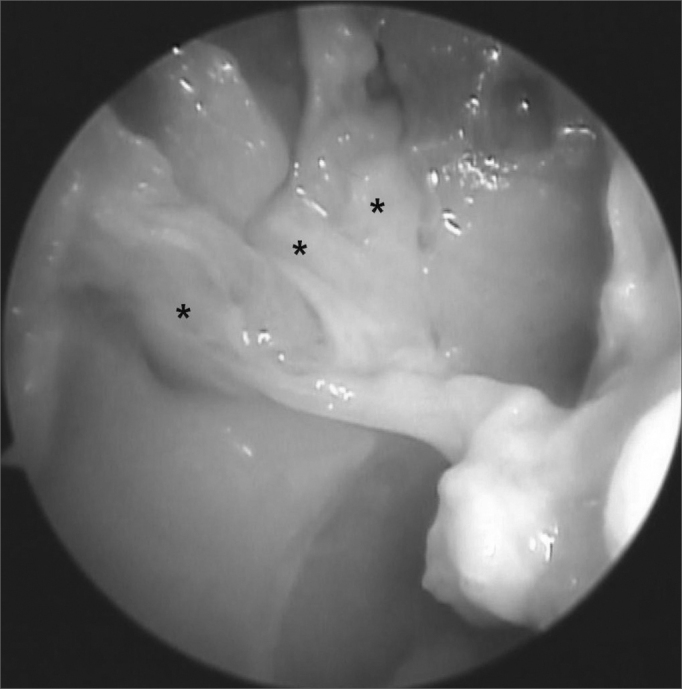
Picture of a right nasal fossa showing three arterial branches (*) emerging from the sphenopalatine foramen.

**Figure 2:**
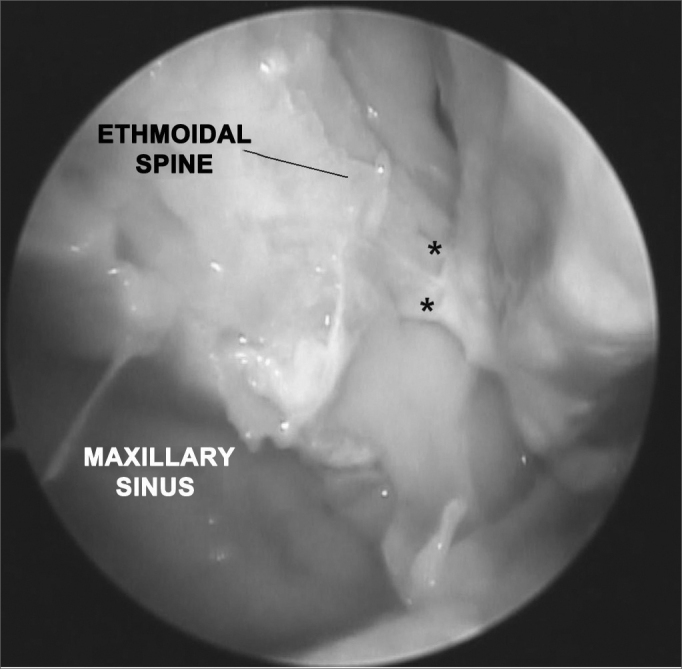
Picture of a right nasal fossa showing two arterial branches emerging from the sphenopalatine foramen. Notice the anatomic correlation between left maxillary sinus, anterior ethmoidal spine, and sphenopalatine foramen.

Most nasal fossae had a single bilateral arterial trunk (n = 12) emerging from the SPF. Others had three (n = 8) or two arterial trunks (n = 5) bilaterally (Figure 1, Figure 2). On only three cases there were variations on the number of branches on each analyzed side. In most cases only one trunk emerged from the accessory SPF. In only two cases two branches were observed on the accessory SPF (Table 4).

**Table 4:** Number of arterial trunks in the sphenopalatine (SPF) and accessory (ASPF) foramens.

Number of arterial branches in the SPF	n	%
One, Bilateral	12	42.8
Two, Bilateral	5	17.8
Three, Bilateral	8	28.6
One on the left, three on the right	1	3.6
Three on the left, two on the right	2	7.2
Number of arterial branches in the SPF	N	%
One, Bilateral	3	25.0
One on the left	3	25.0
One on the right	2	16.7
Two, Bilateral	2	16.7
Not identified	2	16.7

Histology analysis confirmed that all specimens collected were of arterial origin.

**DISCUSSION**

Knowledge on the anatomy of the sphenopalatine artery and its branches is fundamental in the endoscopic treatment of severe posterior epistaxis. Success rates can be greater than 95% in the hands of experienced surgeons. This procedure is associated with low complication rates^7^. However, anatomic variations in the nasal cavity may hamper the exposure of the main and accessory branches of the sphenopalatine artery. Ideally, anatomic landmarks should be identified to make it easier for the surgeon to find these structures. Studies on nasal anatomy done on cadavers or dry skulls aim at identifying these anatomic landmarks and their possible variations.

The methods employed in nasal anatomy studies vary depending on the specimens and approaches used. Herrera Tolosana et al.^8^ did a descriptive study using 32 half-skulls without using endoscopes. Shires et al.^9^ showed a number of anatomic landmarks from 45 heads sectioned sagittally using a zero-degree scope and a videocamera system. In most of their papers, Navarro et al.^10^ make reference to distances between the SPF and various other nose anatomic landmarks using half-skulls and cadaver heads through direct observation with the naked eye. Our study opted to use endoscopy equipment, as this is also an educational opportunity for participating residents and ENT physicians.

Age ranges are rarely cited in studies on nasal anatomy. Cranial facial development from childhood to adult age alters the ratio between the surgical landmarks of the skull^11^. This study included only two young cadavers, one aged 11 and another 15, and did not allow for consistent osteological analysis. Studies with larger samples focused on analyzing age ranges may describe more accurately the differences in the relative positions of the endonasal structures, thus greatly aiding in the treatment of vascular tumors and other pediatric nasal comorbidities^12^.

The ethmoidal spine is present and close to the sphenopalatine foramen in most human beings. Many authors consider the ethmoidal spine as the main anatomic landmark when facing difficulties ligating the sphenopalatine artery during an episode of epistaxis^10^, ^13^. Bolger et al.^13^ studied 22 cadavers and observed that 21 specimens had the ethmoidal spine positioned anteriorly to the SPF, and only one in which it was positioned 3 mm below the SPF. Likewise, Pádua & Voegels^3^ showed that in 98.4% of the cases they analyzed the ethmoidal spine was located anteriorly in relation to the sphenopalatine foramen. Most of the cases included in our study (86%) had the ethmoidal spine located anteriorly in relation to the sphenopalatine foramen. However, in one case the ethmoidal spine was located superiorly to the SPF and in another case it was found posteriorly to the SPF. In one cadaver the ethmoidal spine was absent bilaterally, and in another cadaver it was present only on the left. Despite these variations, the ethmoidal spine was considered an important anatomic landmark in the treatment of severe epistaxis episodes.

Herrera Tolosana et al.^8^ studied 32 half-skulls and accurately described the location of the SPF. In their sample, the most superior portion of the SPF was located at the same level as the higher portion of the choana in 62.5% of the cases. The other 32.5% had the medial portion of the SPF on the same level as the higher portion of the choana. Scanavini et al.^14^ described the location of the SPF in the superior meatus in 81.4% of the cases. Other locations were between the middle and superior meatus in 14.8% of the cases, in the middle meatus in one case, and one case in which the SPF was not found. In our analysis, the SPF was found in the transition between the middle and superior meatus in all cases. This is the most frequently observed location of the SPF as supported by other studies^3^, ^15^. The location of the SPF is harder to be defined in fresh cadavers than in dry half-skulls. Dry specimens allow for increased accuracy in measuring borders and bone foramens when compared to fresh cadavers with preserved nasal mucosas and bleeding vessels.

Simmen et al.^16^ looked into the quantity and location of branches emerging from the SPF and reported that most specimens had two to three arterial branches, and as many as 10 as seen in one case. The authors also explained that the branches were distributed in a superior and inferior position in relation to the ethmoidal spine, and that it was not possible to compare sides as the specimens had come from different heads. Similarly, Babin et al.^17^ reported that the presentation with two or three branches was the commonest, and described a case in which five branches were observed. Eighteen cadavers in our study had more than one arterial branch. Two of them had one more branch on the left than on the right side. Only the complete exposure of the mucosa adjacent to the SPF allows for the visualization of all emerging arterial branches. Thus, mastery of the SPF dissection technique may determine the success of the surgical treatment of severe epistaxis.

The presence of an accessory foramen on the lateral nasal wall has been well established in the literature. However, its occurrence varies significantly. The presence of an accessory foramen may be related to failure of the endoscopic treatment of severe epistaxis^18^. Pádua & Voegels^3^ found an accessory foramen in only 12 (10%) subjects. In our study, 12 (43%) cadavers had an accessory foramen. Our results are similar to those reported by Herrera Tolosana et al.^8^, in which accessory foramens were found in 16 (50%) of their cases, six with two orifices and ten with only one. In terms of accessory foramen location, Herrera Tolosana et al.^8^ reported that most of them are in the middle meatus (73%) right below the SPF. We have also observed that smaller bone orifices in the point of emergence of arterial branches other than the main sphenopalatine foramen were mostly located in the middle meatus. In most of the cases only one accessory foramen was observed. Navarro et al.^10^ believed that one of these accessory branches could be the posterior lateral nasal artery or other arterial branches. We understand that reaching consensus as to the name of these accessory branches can be a complex task, given the anatomical variations of these vessels. The simple knowledge on the possible presence of these accessory foramens during nasal endoscopic surgery may prevent maneuvers and ill-advised dissections on the lateral nasal wall, reducing morbidity and the time our patients spend on general anesthesia.

**Figure 3:**
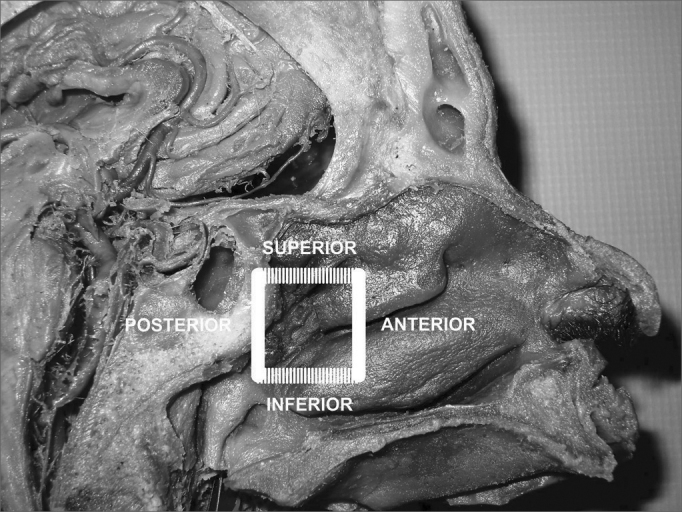
Picture of a skull sagittal view showing the left nasal fossa and the ipsilateral sphenopalatine artery. Knowledge on the limits of the “sphenopalatine quadrangle” may aid surgeons identifying all branches and foramens of the sphenopalatine artery.

**Figure 4:**
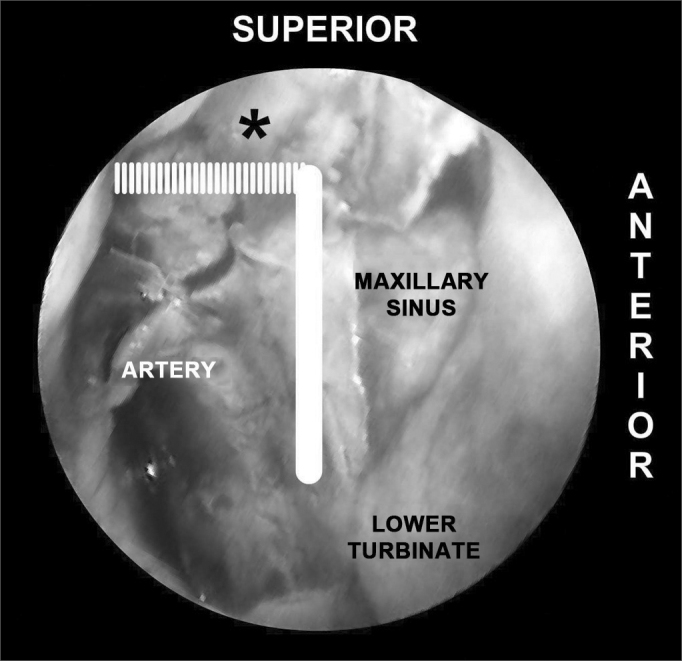
Picture of a left nasal fossa showing details of the “sphenopalatine quadrangle.” In the superior border, the tip to find the accessory foramens is the fat tissue from the pterygopalatine fossa (*). The perpendicular portion of the palatine bone is the anterior border and the starting point from which the mucoperiosteal flap is produced to find the sphenopalatine foramen.
• SUPERIOR: the highest point of the anterior plate of the palatine bone ends where it meets the fat tissue of the pterygopalatine fossa. The dissection of the mucoperiosteal flap up to this level will reveal all possible accessory foramens of the superior portion of the sphenopalatine artery complex (Figure 4).
• INFERIOR: the insertion point of the inferior concha is the most inferior point in the extension of the mucoperiosteal flap. From this line the accessory branches of the sphenopalatine artery cannot be identified (Figure 5).

In order to ensure the identification of all branches of the sphenopalatine artery, we propose the delimitation of the endoscopic “Sphenopalatine Quadrangle” (Figure 3) for the blood vessels of the posterior nasal wall. The tips of this area match the borders of a quadrangle with the following names:

• ANTERIOR: the anterior border of the perpendicular portion of the palatine bone is the best landmark to start dissecting the mucoperiosteal flap. The easiest way to find this landmark is by palpating the posterior medial maxillary wall at the level of the hrizontal portion of the middle concha. The posterior medial maxillary wall is a moving soft structure. As you palpate it from the anterior to the posterior nasal fossa, you will soon find a stiff hard structure, the anterior plate of the palatine bone, where the mucoperiosteal detachment is initiated (Figure 3, Figure 4).

**Figure 5:**
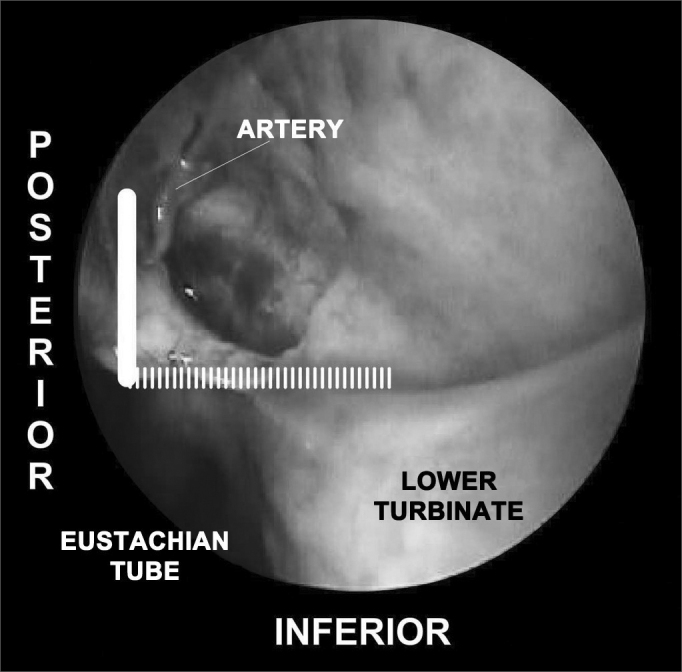
Picture of a left nasal fossa. The inferior border of the “sphenopalatine quadrangle” is the superior portion of the inferior turbinate. The Eustachian tube is the posterior border of the mucoperiosteal flap.
• POSTERIOR: as the anterior wall of the sphenoid sinus and the Eustachian tube are identified, the dissection of the mucoperiosteal flap is completed with the certainty that all branches and foramens of the sphenopalatine artery have been identified (Figure 5).

## CONCLUSION

The anatomy of the sphenopalatine artery is highly variable. The ethmoidal spine is an important anatomic landmark, as it is present in almost all cases in a position anterior to the sphenopalatine foramen. The most frequent location of the sphenopalatine foramen was in the transition between the middle and superior meatus.
